# Stressful life events increase the risk of major depressive episodes: A population-based twin study

**DOI:** 10.1017/S0033291722002227

**Published:** 2023-08

**Authors:** Ludvig D. Bjørndal, Kenneth S. Kendler, Ted Reichborn-Kjennerud, Eivind Ystrom

**Affiliations:** 1 PROMENTA Research Center, Department of Psychology, University of Oslo, Oslo, Norway; 2 Department of Mental Disorders, Norwegian Institute of Public Health, Oslo, Norway; 3 School of Pharmacy, University of Oslo, Oslo, Norway; 4 Department of Psychiatry, Virginia Commonwealth University School of Medicine, Richmond, VA, USA; 5 Department of Human and Molecular Genetics, Virginia Commonwealth University School of Medicine, Richmond, VA, USA; 6 Institute of Clinical Medicine, University of Oslo, Oslo, Norway

**Keywords:** Co-twin control, depression, stressful life events, survival analysis, twin study

## Abstract

**Background:**

Previous studies have found that stressful life events (SLEs) are associated with an increased risk of adult depression. However, many studies are observational in nature and limited by methodological issues, such as potential confounding by genetic factors. Genetically informative research, such as the co-twin control design, can strengthen causal inference in observational studies. Discrete-time survival analysis has several benefits and multilevel survival analysis can incorporate frailty terms (random effects) to estimate the components of the biometric model. In the current study, we investigated associations between SLEs and depression risk in a population-based twin sample (*N* = 2299).

**Methods:**

A co-twin control design was used to investigate the influence of the occurrence of SLEs on depression risk. The co-twin control design involves comparing patterns of associations in the full sample and within dizygotic (DZ) and monozygotic twins (MZ). Associations were modelled using discrete-time survival analysis with biometric frailty terms. Data from two time points were used in the analyses. Mean age at Wave 1 was 28 years and mean age at Wave 2 was 38 years.

**Results:**

SLE occurrence was associated with increased depression risk. Co-twin control analyses indicated that this association was at least in part due to the causal influence of SLE exposure on depression risk for event occurrence across all SLEs and for violent SLEs. A minor proportion of the total genetic risk of depression reflected genetic effects related to SLEs.

**Conclusions:**

The results support previous research in implicating SLEs as important risk factors with probable causal influence on depression risk.

## Introduction

Stressful life events (SLEs) are environmental factors that increase the risk of depression (Kessler, 1997; Tennant, 2002). Many different events can be classified as stressful, such as experiencing assault, job loss or sexual abuse. All of these SLEs have been implicated as risk factors for depression in genetically informative studies (Kendler et al., 1995, 2000; Kendler & Aggen, 2014; Kendler & Gardner, 2001; Kendler, Karkowski, & Prescott, 1999a). SLEs that occur during childhood, adolescence and adulthood are all associated with an increased risk of depression (Sjöholm, Lavebratt, & Forsell, 2009; Tennant, 2002).

Research on exposure to risk factors for illness is typically observational in nature due to ethical or practical constraints. Many observational studies are limited by methodological issues, such as potential confounding by genetic or environmental factors and reporting bias. Genetically informative samples can strengthen causal inference in observational research (Pingault et al., 2018). The co-twin control design focuses on twins discordant for exposure to risk factors or outcomes and is one strategy for increasing control over potential confounders.

Findings from several genetically informative studies have indicated that the associations between SLEs and depression in part reflect the causal influence of exposure on depression risk (Kendler et al., 1999a; Kendler & Aggen, 2014; Kendler & Gardner, 2010). However, it has been found that associations may in part reflect non-causal influence or confounding by genetic or familial factors (Brown et al., 2014; Kendler et al., 1999a; Silberg et al., 2016). These conflicting findings warrant further studies of the relationship between SLE exposure and depression risk with genetically informative samples. Attaining a better understanding of the nature of the influences of SLE exposure on depression risk can have implications for both efforts to prevent depression and for clinical practice.

In the current study, we investigated associations between SLEs and depression risk in a population-based sample of twins (*N* = 2299). Three primary research questions were examined: (a) Is the occurrence of any SLEs, relational violent SLEs (i.e. SLEs for which another person is responsible), non-relational other traumatic SLEs (i.e. SLEs for which another person is not necessarily responsible), and/or economic SLEs associated with increased risk of depression?; (b) can indicated causal influence of the different groups of SLEs on depression risk be detected in co-twin control analyses?; and (c) how much of the total genetic risk of depression can be explained by the genetic risk associated with SLEs?

## Methods

### Sample

Participants were recruited from the Norwegian Institute of Public Health Twin Panel (NIPHTP). The NIPHTP has been described at length elsewhere (Harris, Magnus, & Tambs, 2002, 2006; Nilsen et al., 2013). Zygosity was determined by a combination of questionnaire items and genotyping.

Data came from a population-based study of psychiatric disorders among NIPHTP participants with two waves of data collection. From 1999 to 2004 (W1), psychiatric disorders were measured in a subsample of NIPHTP participants. In total, psychiatric interviews were conducted with 2801 twins. Mean age at W1 was 28.2 years (range 19–36). At W1, there were 449 MZ female twin pairs, 220 male MZ twin pairs, 263 female DZ twin pairs, 118 male DZ twin pairs, 341 DZ pairs of the opposite sex and 19 single twins.

Wave 2 (W2) of data collection was initiated in 2010 (Nilsen et al., 2013). Mean age at W2 was 37.8 years (range 30–44). Clinical diagnostic interviews were completed on 2284 of the twins interviewed during W1. At W2, there were 358 female MZ twin pairs, 154 male MZ twin pairs, 180 female DZ twin pairs, 76 male DZ twin pairs, 219 DZ twin pairs of the opposite sex, and 310 single twins. Data on occurrences of SLEs was collected using a questionnaire, which was based on a measure which has been used to record SLE occurrences in previous studies (Kendler et al., 1995). In total, 2299 twins responded to the questionnaire at W2. We report additional characteristics of the sample in the online Supplementary Materials.

All participants gave their written informed consent to participate in the study. Approval was sought and received from the Regional Committee for Medical and Health Research Ethics (#2010/767).

### Measurements

Psychiatric interviews were conducted using the Munich-Composite International Diagnostic Interview (M-CIDI; Wittchen and Pfister, 1997). M-CIDI assesses DSM-IV and ICD-10 diagnoses and has good validity and reliability (Reed et al., 1998; Wittchen, Lachner, Wunderlich, & Pfister, 1998). Interviewers were primarily clinical psychology students in the final part of their degree or psychiatric nurses, all of whom attended standardised CIDI training. Interviews were largely conducted in person (8.3% of the interviews were conducted by telephone for practical reasons) in W1. All interviews were conducted via telephone in W2. Prevalence of DSM-IV Major Depressive Episode (MDE) is listed in Table 1.

**Table 1.** Depression diagnoses at Wave 1 and Wave 2

MDE	Wave 1	Wave 2
Subthreshold	138	91
Threshold	411	406
Total	549	497

Subthreshold diagnoses were treated as diagnoses of depression for the primary analyses to maximise statistical power. Subthreshold diagnoses in M-CIDI are assigned when an individual meets all mandatory diagnostic criteria except for one (Wittchen et al., 1998). Analyses with only threshold diagnoses yielded similar results (see online Supplementary Appendix A). MDE diagnoses were recorded per year of each participant's life. The median age of MDE onset in Wave 1 was 21 years. The median age of MDE onset in Wave 2 was 29 years.

The SLE questionnaire included 18 SLEs. If a participant answered yes for any SLE, he or she was asked to enter the age (in years) when the event was experienced (or when it was first experienced if it had occurred multiple times). The frequencies of all 18 SLEs in the sample are listed in Table 2.

**Table 2.** Frequencies of stressful life events

Stressful life events (SLEs)	Frequency in full sample	SLEs before MDE, W1 (Median age)	SLEs before MDE W2 (Median age)
Life-threatening illness	125	7 (12)	20 (17.5)
Life-threatening accident	151	17 (17)	25 (21)
Actively participated in war/combat	31	2 (24)	6 (25)
Witnessed anyone be badly injured or killed	229	17 (16)	34 (23.5)
Threatened with a weapon, held captive or kidnapped	153	15 (19)	29 (21)
Experienced fire, flooding or natural disaster	133	6 (17.5)	23 (21)
Rape	91	19 (16)	26 (13.5)
Sexual abuse	176	51 (10)	56 (10)
Otherwise physically attacked or assaulted	303	42 (16.5)	54 (18)
Otherwise physically abused as a child	76	26 (8)	28 (7.5)
Otherwise mistreated as a child	68	19 (10)	27 (10)
Parental mental illness or alcohol problems as a child	395	105 (4)	110 (5)
Parental divorce or separation as a child	451	85 (11)	89 (10)
Having divorced or separated	709	29 (26)	103 (28)
Long-term financial difficulties	164	14 (21.5)	27 (24)
Unemployment for more than six months	236	17 (20)	35 (24)
Major and lasting conflict with close person	269	21 (20)	40 (25)
Other serious life event	441	35 (16)	70 (24)

*Note.* The table lists the frequency of SLEs in the full sample, SLEs that occurred before a depression diagnosis reported in W1, and SLEs that occurred before a depression diagnosis reported in W2. Median age for SLE occurrence (of time-censored SLEs) is reported in years.

The SLEs were aggregated in four groups for statistical analyses. *All SLEs* included all 18 SLEs listed in Table 2 and represented the overall effect of event exposure across all SLEs. Further grouping of SLEs was based on a distinction between relational violent SLEs (i.e. SLEs for which another person is responsible), non-relational violent SLEs, and economic or financial SLEs. The SLEs of the current study did not fit neatly into existing typologies such as the independent/dependent SLE categorisation (Kendler, Karkowski, & Prescott, 1999b). Distinctions between relational and non-relational SLEs and economic SLEs have been used in some previous studies (Anders, Shallcross, & Frazier, 2012; Stone et al., 2015; Ward, Kanu, & Robb, 2017).

*Relational violent SLEs* included five SLEs: threatened with a weapon, held captive or kidnapped; sexual abuse; rape; otherwise physically attacked or assaulted; and otherwise physically abused as a child. *Non-relational other traumatic SLEs* included three SLEs: life-threatening accident; actively participated in war/combat; and witnessed anyone be badly injured or killed. *Economic SLEs* included two SLEs: long-term financial difficulties and unemployment for more than six months.

### The biometric model and the co-twin control design

Twin designs are based on the varying genetic relatedness between monozygotic (MZ) twins, who share 100% of their genetic material, and dizygotic (DZ) twins, who share 50% of their genetic material. The basic notion is that, if genetic influences are important for variation in a phenotype, MZ twins will be (on average) more similar than DZ twins for the given phenotype. The biometric model explains phenotypic variation as influenced by additive genetic factors (A), non-additive genetic effects (D), shared environmental factors (C) and non-shared environmental factors (E) (Eaves, Last, Young, & Martin, 1978; Fisher, 1918; Jinks & Fulker, 1970).

The co-twin control design represents one approach to studying exposure to environmental factors that cannot be experimentally manipulated using twin data. The design compares associations in the full sample and for discordant twins. As MZ and DZ twins are matched for the early shared environment, and either partially (DZ) or fully (MZ) for genetic material, this increases control over potential confounding by these factors. The co-twin control design can be understood within a counterfactual model of causality (McAdams, Rijsdijk, Zavos, & Pingault, 2021).

Patterns of associations in the full sample and in within-pair estimates for MZ and DZ twins help discern if environmental exposure may have a causal influence on the outcome. If observed associations are equally strong in the full sample and for discordant DZ and MZ twins, this is indicative of causal influence, as within-pair estimates control for shared environmental and genetic factors (partially for DZ twins and fully for MZ twins). Weaker associations within twins compared with the full sample are indicative of confounding by early shared environment. If the associations weaken with genetic similarity (i.e. strongest in the full sample and weakest in MZ twins), this is indicative of genetic confounding, which is controlled for to a varying extent in within-pair estimates.

### Discrete-time survival analysis

Associations were modelled using discrete-time survival analysis. Survival analysis has several benefits, including the opportunity to incorporate right-censored observations (i.e. when the time to event is larger than the period of data collection) in estimates of event probabilities and study how the risk of events of interest is distributed over time (Hox, 2010). In the discrete-time survival model, a hypothetical coefficient *β*_1_ can be interpreted as the change in the logit of the hazard, following a one unit increase in the corresponding covariate. This effect is independent of specific time intervals. Multilevel survival models can incorporate random effects (often called *frailty*; Austin, 2017).

### Statistical analyses

Analyses were conducted using discrete-time survival analysis with frailty terms to estimate the components of the biometric model. The dataset was converted to a ‘long format’, which is required for survival analysis (Hox, 2010). SLEs that had occurred after the onset of a depression diagnosis were time-censored (i.e. given the value of 0), so that only SLEs that occurred before MDE diagnoses were included in analyses. Only first onset MDE diagnoses for each interview were used in the analyses (i.e. only the first diagnosis was included in the analysis if an individual reported multiple depressive diagnoses, but participants were allowed to report different depressive episodes in W1 and W2).

A parameterisation for between- and within-family effects proposed by Rabe-Hesketh, Skrondal, and Gjessing (2008) was applied to estimate the biometric components, with some modifications. Random effects were added at three levels: A random slope at the level of the twin pairs, shared by all twin pairs; a random slope at the level of ‘zygosity’, shared only by MZ twins; and one random effect at the level of participants, capturing residual dependence between measures from the same individual. The variables estimated as random slopes were given the value of 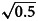. This parameterisation ensures that the co-variance for MZ and DZ twins differs depending on the type of twin.

The described model estimates the AE components. The ACE model was estimated by including an additional random intercept capturing residual dependence between individuals within the same twin pair not captured by genetic effects. The CE model was estimated by including the additional intercept and dropping the Level 2 and Level 3 random effects. The ADE model was not estimated as previous studies have not found evidence of non-additive genetic effects for depression (Kendler & Prescott, 1999; Kendler, Neale, Kessler, Heath, & Eaves, 1992).

Two constraints were used in the model. The first constrained the residual variance of the random slopes representing genetic effects at Level 2 and Level 3 to be equal. The second constrained the surplus within and the ‘sparsity’ between fixed effects of the DZ twins to be the inverse of each other (these can be constrained to be the inverse of each other as both represent the effects of 0.5A).

The random effects were added in a generalised linear mixed model (GLMM) with a binomial distribution and a *logit* link function. The variance of the random effects can be compared against the residual variance (or error) at the lowest-level, which is (*π*^2^)/3 for the logistic distribution (Hox, 2010). This allows for estimating the biometric components. The intraclass correlation for the models can then be calculated using the estimates of the random effects, i.e., the between estimate divided by the total variance, which is the sum of the between estimate plus the residual variance.

Age of onset was fitted as a fixed effect and inspected up to the third order polynomial, after which it did no longer contribute to model fit. Age of onset, sex and wave of measurement (W1 or W2) were included as fixed effects. The dependent variable was having received a depression diagnosis at any time point. Models were evaluated using AIC (Akaike, 1987). The ACE model did not converge to estimate a confidence interval for the intercept estimating the C component (*AIC* = 8715.203), likely because this variance parameter was very close to 0 or negative. The C component could be dropped without deterioration in model fit for the AE model (*AIC* = 8715.203). The CE model did not improve model fit (*AIC* = 8719.587). Therefore, the AE model served as the basis for subsequent models that incorporated the effects of SLEs.

## Results

### Model with no SLEs

Table 3 reports the results for the fixed and random effects in the AE model with no SLEs.

**Table 3.** Fixed and random effects of the AE model

Fixed part	*β*	s.e.	*p*	95% Confidence interval
Age of onset (*t*)	0.462	0.084	<0.001	[0.297–0.627]
Age of onset^2^	−0.011	0.004	0.003	[−0.018 to −0.004]
Age of onset^3^	0.000	0.000	0.058	[−0.000 to 0.000]
Wave 2	−0.572	0.086	<0.001	[−0.741 to −0.404]
Sex (female)	0.632	0.122	<0.001	[0.393–0.870]
Fixed intercept	−11.138	0.637	<0.001	[−12.386 to −9.890]
Random part				
σ_A_	1.330	0.245		[0.926–1.909]
σ_E_	0.946	0.260		[0.552–1.621]

The fixed effects of time *t* determine the baseline hazard rate, i.e., the hazard when all covariates are zero (Rabe-Hesketh & Skrondal, 2008). Risk of depression increased over time but was slightly reduced at later time points (i.e. older ages) in the sample. The hazard rate peaked around age 30 and depression risk was higher for females.

The heritability estimate for receiving a first subthreshold or threshold MDE diagnosis in any given year was 24%. By excluding the lowest-level error term, the heritability of lifetime risk of depression was estimated at 58%. This latter approach is asymptotic to the ‘measurement model’ of psychometrics, where random measurement error is excluded from effect estimates (Kendler, Neale, Kessler, Heath, & Eaves, 1993; van den Berg, Glas, & Boomsma, 2007).

Stable and unstable environmental effects on depression risk were disentangled by dividing the estimated *σ*_*E*_ and the lowest-level variance by the total variance. In any given year in the current sample, depression risk was explained by 24% genetic influences, 17% stable (time-invariant) environmental influences, and 59% unstable (time-variant) environmental influences including measurement error.

### Co-twin control analyses

Table 4 reports the results of co-twin control analyses of associations between grouped SLEs and depression risk.

**Table 4.** Results of co-twin control analyses displaying hORs

	Full sample			Within DZ twins			Within MZ twins		
SLEs	hOR (s.e.)	95% CI	*p*	hOR (s.e.)	95% CI	*p*	hOR (s.e.)	95% CI	*p*
All	1.383 (0.046)	[1.296–1.475]	<0.001	1.398 (0.151)	[1.131–1.727]	0.002	1.423 (0.114)	[1.216–1.664]	< 0.001
Violent	1.551 (0.114)	[1.343–1.790]	<0.001	1.310 (0.333)	[0.797–2.155]	0.287	1.430 (0.238)	[1.032–1.982]	0.032
Other traumatic	1.176 (0.172)	[0.883–1.567]	0.267	0.711 (0.336)	[0.282–1.793]	0.470	0.784 (0.245)	[0.425–1.445]	0.436
Economic	2.021 (0.291)	[1.524–2.681]	<0.001	3.307 (1.823)	[1.122–9.744]	0.030	3.103 (1.043)	[1.606–5.995]	0.001

*Note.* hOR refers to hazard odds ratio (Petras et al., 2011). Associations were statistically significant if corresponding *p* values were below a threshold of *α* = 0.05

The occurrence of one SLE (across all 18 SLEs) was associated with increased depression risk in the full sample. The associations were similar in size for DZ twins and MZ twins.

For violent SLEs, the occurrence of one SLE was associated with increased depression risk in the full sample and for MZ twins. The within-pair association for DZ twins was slightly attenuated compared with the within-pair estimate for MZ twins and was not statistically significant. For other traumatic SLEs, the occurrence of one SLE was not associated with an increased risk of depression in the full sample, nor for MZ twins or DZ twins. For economic SLEs, the occurrence of one event was associated with increased depression risk in the full sample, and stronger associations were observed for MZ twins and for DZ twins. The time-censoring means that the SLEs predicted future depression risk – no backward associations could occur.

Table 5 displays the residual random effects estimated in the models incorporating the various SLEs. Heritability was estimated before and after controlling for SLEs. The reduction in *σ_A_* after including SLEs reflects how much of the total genetic risk of depression which was explained by genetic effects linked to SLEs, in the present sample. After including all of the SLEs, the heritability of depression went from 58% to 50%.

**Table 5.** Residual random effects for the models with grouped SLEs

Grouped SLEs	*σ*_*A*_	95% CI (*σ*_*A*_)	*σ*_*E*_	95% CI (*σ*_*E*_)	Residual *h*^2^
Baseline model	1.330	[0.926–1.909]	0.946	[0.552–1.621]	0.584
All SLEs	1.031	[0.666–1.596]	1.039	[0.623–1.732]	0.498
Violent SLEs	1.152	[0.777–1.709]	0.928	[0.541–1.593]	0.554
Other traumatic SLEs	1.307	[0.903–1.890]	0.966	[0.566–1.649]	0.575
Economic SLEs	1.239	[0.848–1.810]	0.949	[0.554–1.624]	0.566

*Note*. Table 5 displays residual random effects in the AE models incorporating the effects of SLEs and the reduction in additive genetic effects after including SLEs in the models (in percentages). These were compared to the random effects estimated in the AE model with no SLEs, listed in Table 3.

## Discussion

Overall, SLE occurrence was associated with an increased risk of depression for all 18 SLEs, violent SLEs and economic SLEs in the full sample. For all SLEs and violent SLEs, co-twin control analyses indicated that these associations at least in part reflect the causal influence of SLE exposure on depression risk. The occurrence of other traumatic SLEs was not associated with increased depression risk. A small proportion of the genetic risk of depression reflected genetic effects linked to SLEs.

The results of our co-twin analyses of the occurrence of all SLEs and violent SLEs are in accordance with previous genetically informative studies that have found that the association between dependent SLEs and depression risk is likely substantially causal in nature (Kendler et al., 1999a; Kendler & Gardner, 2010). Almost all events in the current study were personal SLEs (i.e., SLEs that occur to individuals, in contrast to SLEs occurring to people within an individual's social network): indicated causal influence of exposure to personal SLEs on depression risk has also been found previously (Kendler et al., 1999a). We find that occurrences of violent SLEs increase depression risk when controlling for shared genetic and environmental factors, which aligns with previous studies which have assessed associations between various forms of trauma and depression risk (Brown et al., 2014; McCutcheon et al., 2009).

Causal influence on the risk of depression has been suggested for a number of specific SLEs included in the current study, such as assault (Kendler et al., 1999a, 2010), sexual abuse (Kendler et al., 2000; Kendler & Aggen, 2014; Nelson et al., 2002), and romantic problems and job loss (Kendler & Gardner, 2001). Our results are in agreement with the findings of with these previous studies, although we analysed aggregated SLEs. Evidence of a causal effect of maltreatment in childhood on the risk of major depression was also found in a recent genome-wide association study (GWAS) meta-analysis using Mendelian Randomisation (Warrier et al., 2021). Some SLEs, like sexual abuse, affect women and men with different frequencies (Barth, Bermetz, Heim, Trelle, & Tonia, 2013).

No associations between the occurrence of other traumatic SLEs (i.e. life-threatening accident, participated in the war, witnessed anyone be badly injured or killed) and risk of depression were observed in the current study. This is not in accordance with results from previous genetically informative studies, which have found increased depression risk associated with combat exposure (Koenen et al., 2003) and somatic illness and injury (Kendler et al., 1999a). While it is not clear why there is an absence of associations between other traumatic SLEs and depression risk in the current study, we note that this aggregate included fewer SLEs compared with violent and all SLEs and was substantially more heterogeneous compared with the other aggregated SLEs.

For economic SLEs, strong associations were observed between event occurrence and increased risk of depression in the full sample. This is in line with previous genetically informative studies that have found increased depression risk associated with financial problems and job loss (Kendler et al., 1999a; 2010; Kendler & Gardner, 2001; 2014).

However, observed associations between economic SLEs and risk of depression were stronger in within-pair estimates for MZ and DZ twins compared with the full sample. It is not clear why these results were found for economic SLEs. Two possible explanations are proposed. Firstly, it may be the case that this association was suppressed in the full sample due to confounding. This could have resulted if there was an inverse effect of the confounder(s) on exposure and outcome, which masked or suppressed an effect in the full sample (e.g. if familial factors associated with less economic problems were associated with increased depression risk, this could suppress the full sample association).

A second possible explanation is that the within-pair associations were inflated due to non-shared confounders in twin pairs. Stronger associations in within-pair estimates than the full sample can result when confounding variable(s) within twin pairs have lower correlation than the correlation of exposure to a risk factor within twin pairs (Frisell, Öberg, Kuja-Halkola, & Sjölander, 2012). As discordant twins are selectively chosen to be dissimilar, this implies that they differ in non-shared causes of exposure to risk factors (i.e. a twin pair discordant for exposure is likely less similar than a twin pair concordant for exposure). If a confounder increases the probability of exposure to economic SLEs and depression, an unexposed twin would be less likely to possess the confounder and become depressed, as the confounder increases the risk of depression. This can potentially inflate within-pair estimates.

Several previous genetically informative studies have implicated economic SLEs as risk factors for depression, but not specifically reported on within-pair estimates of the associations (Kendler et al., 1999a, 2010; Kendler & Halberstadt, 2013). Although the inflated within-pair estimates in our study are difficult to interpret, findings from some previous genetically informative studies indicate that this association may be at least in part causal. Using a similar design as in the current study, Kendler and Gardner (2001) found that MZ twins with a history of depression reported higher rates of job loss compared with non-depressed co-twins. Kendler and Gardner (2014) found that the association between financial and occupational difficulties and depression risk were stronger in male twins discordant for depression compared with female twins.

### Limitations

The current study has some potential limitations. Although the co-twin control design increases control over genetic and shared environmental confounders, it is unable to control for non-shared confounders that only affect one twin, which can bias estimated effects (Frisell et al., 2012; Kendler & Gardner, 2010). We focused on the main effects of SLEs. Previous studies have, however, found genotype-environment interactions for SLEs and depression (e.g. Choi *et al*. 2020; Mandelli & Serretti, 2013). The current study was retrospective in nature. Therefore, the possible influence of recall bias (i.e. that current depression or mood states affects correct reporting of previously experienced SLEs or depressive episodes) cannot be excluded. Participants were censored after the first onset of a MDE, and so we did not study the effects of SLEs on recurrent depression. While we adjusted for sex in the statistical models, we did not investigate if there were different causes of life events and depression by gender. Previous studies have indicated that men and women share most, but not all, genetic factors that influence the risk of depression (Kendler, Ohlsson, Lichtenstein, Sundquist, & Sundquist, 2018; Sullivan, Neale, & Kendler, 2000). Similarly, one previous study found a substantial genetic correlation between men and women for genetic factors influencing the propensity to experience personal SLEs (Bolinskey, Neale, Jacobson, Prescott, & Kendler, 2004). A number of limitations apply to our measurement of SLEs. Several SLE categories were relatively broad, e.g., ‘sexual abuse’, and may therefore capture events with different relationships to depression risk. For instance, it has been found that the risk of psychiatric disorders is higher for childhood sexual abuse that involves intercourse compared with nongenital sexual abuse (Kendler et al., 2000). Most studies have found that SLEs primarily increase depression risk over a shorter time period (e.g. months; Kendler, Karkowski, & Prescott, 1998, 1999a). Although SLEs were time-censored so that only SLEs occurring before depression were included in analyses, SLE occurrence and MDE onset were recorded to the nearest year. Thus, the effects could have been different if the timing of SLE occurrence was recorded closer to the MDE onset. In addition, our analyses could have included SLEs with long timespans between SLE occurrence and MDE onset (as long as the SLE occurred before the diagnosis), and the relationship between SLE exposure and depression onset with long time intervals is less clear. Additional descriptive analyses (see online Supplementary Appendix B) where we investigated the temporal relationship between SLE occurrence and MDE onset align with the previous literature, suggesting that most SLEs occur the same year as MDE onset. Additionally, previous studies have shown that high long-term contextual threat increases MDE onset following SLE occurrence substantially (Kendler et al., 1998), but we did not assess long-term contextual threat in the current study. We assessed SLEs using a questionnaire and not, for instance, using trained interviewers. This could potentially result in higher inter-respondent variation in interpretation of the 18 SLEs (e.g. for sexual abuse), which could result in either under-reporting or over-reporting.

### Implications

The findings of the current study have several implications. The results support previous research in implicating SLEs as important risk factors with probable causal influence on depression risk. Some of the SLEs included in our study (e.g. job loss) are indirectly influenced by policy and our findings underline the importance of preventing SLEs, when possible. The influence of SLEs on depression risk also suggests that trauma could be a useful focus in psychological treatments.

When controlling for SLE occurrence (across all 18 SLEs), the estimated heritability of depression was reduced by 8%, indicating that a minor proportion of the genetic risk of depression reflected genetic effects linked to SLEs. This estimate aligns with that of one previous study, which found that roughly 10–15% of the impact of genetic risk of depression was mediated through life experiences (Kendler & Karkowski-Shuman, 1997). Minor genetic effects linked to SLEs suggest that it is unlikely that a large proportion of identified genetic variants will be linked to SLEs in GWASes. Our study also illustrates the utility of quantitative genetic designs in estimating genetic factors with direct effects and genetic factors acting through a heritable exposure, as we find that a minor proportion of genetic effects on depression risk reflect genetic effects related to SLE exposure. The heritability estimate for one-year depression risk was slightly lower than the estimate reported by Kendler and Gardner (2017), but close to heritability estimates for depressive symptoms in the past year (Matthews et al., 2016) and in the past two weeks (Czajkowski, Røysamb, Reichborn-Kjennerud, & Tambs, 2010).

## Conclusion

In the current study, we investigated patterns of associations between exposure to SLEs and depression risk in a large population-based twin sample using a co-twin control design. Diagnoses of depression were based on repeated clinical interviews across two waves of measurements. Event occurrence across all 18 SLEs, of violent SLEs and of economic SLEs was associated with an increased risk of depression. For the former two, co-twin control analyses indicated that the associations at least in part reflect the causal influence of SLE exposure on depression risk. The pattern of associations for economic SLEs could have resulted from suppression of the full sample association or inflated within-estimates due to non-shared confounding. No associations between other traumatic SLEs and depression risk were observed. A minor proportion of the total genetic risk of depression reflected genetic effects related to SLEs.

## Supplementary material

For supplementary material accompanying this paper visit https://doi.org/10.1017/S0033291722002227. click here to view supplementary material
