# Supplementary material for: Stressful life events increase the risk of major depressive episodes: A population-based twin study
Source: Psychol Med. 2022 Aug 3;53(11):5194–202. doi: 10.1017/S0033291722002227 (PMC10476058; doi:10.1017/S0033291722002227)
Supplement: Supplementary file 1 [file S0033291722002227sup001.docx]

**Supplementary material**

**Appendix A**

Appendix A reports the output for models run with only threshold MDE diagnoses. These yield very similar estimated associations as the models reported in the article (which used both subthreshold and threshold diagnoses).

**Table A1**

*AE Model With All 18 SLEs and Only Threshold MDE Diagnoses*

| Fixed part | β | *SE* | p | 95% Confidence Interval |  |  |
| --- | --- | --- | --- | --- | --- | --- |
| SLE-total [cluster mean] | .368 | .046 | < .001 | [.278, .458] | | |
| SLE-tot [cluster mean] * dizygotic | .011^a^ | .052 | .838 | [-.092, .113] | | |
| SLE-total [cluster mean centered] | .330 | .077 | < .001 | [.180, .481] | | |
| SLE-total [cluster mean centered] * dizygotic | -.011^a^ | .052 | .838 | [-.113, .092] | | |
| Age of onset (*t*) | .444 | .087 | < .001 | [.274, .614] | | |
| Age of onset^2^ | -.012 | .004 | .001 | [-.019, -.004] | | |
| Age of onset^3^ | .000 | .000 | .029 | [.000, .000] | | |
| Wave 2 | -.422 | .085 | < .001 | [-.590, -.255] | | |
| Sex (female) | .634 | .122 | < .001 | [.395, .873] | | |
| Fixed intercept | -11.644 | .676 | < .001 | [-12.969, -10.319] | | |
| Random part |  |  |  |  |  |  |
| $\sigma_{A}$ | 1.235 | .268 |  | [.807, 1.889] | | |
| $\sigma_{E}$ | 1.220 | .299 |  | [.754, 1.973] | | |

AIC = 8788.451 (df = 11)

^a^Estimated as single parameter.

**Table A2**

*AE Model With Violent SLEs and Only Threshold MDE Diagnoses*

| Fixed part | β | *SE* | p | 95% Confidence Interval |  |  |
| --- | --- | --- | --- | --- | --- | --- |
| Violent SLE [cluster mean] | .569 | .109 | < .001 | [.355 .784] | | |
| Violent SLE [cluster mean] * dizygotic | -.014^a^ | .130 | .914 | [-.269, .241] | | |
| Violent SLE [cluster mean centered] | .328 | .155 | .035 | [.023, .632] | | |
| Violent SLE [cluster mean centered] * dizygotic | .014^a^ | .130 | .914 | [-.241, .269] | | |
| Age of onset (*t*) | .444 | .086 | < .001 | [.276, .613] | | |
| Age of onset^2^ | -.011 | .004 | .002 | [-.018, -.004] | | |
| Age of onset^3^ | .000 | .000 | .031 | [.000, .000] | | |
| Wave 2 | -.405 | .085 | < .001 | [-.571, -.238] | | |
| Sex (female) | .633 | .122 | < .001 | [.394, .872] | | |
| Fixed intercept | -11.578 | .671 | < .001 | [-12.892, -10.263] | | |
| Random part |  |  |  |  |  |  |
| $\sigma_{A}$ | 1.336 | .269 |  | [.900, 1.981] | | |
| $\sigma_{E}$ | 1.130 | .287 |  | [.687, 1.859] | | |
|  |  |  |  |  | | |

AIC = 8863.352 (df = 11)

^a^Estimated as single parameter.

**Table A3**

*AE Model With Other Traumatic SLEs and Only Threshold MDE Diagnoses*

| Fixed part | β | *SE* | p | 95% Confidence Interval |  |  |
| --- | --- | --- | --- | --- | --- | --- |
| Other traumatic SLE [cluster mean] | .654 | .196 | .001 | [.269, 1.039] | | |
| Other traumatic SLE [cluster mean] * dizygotic | -.085^a^ | .240 | .724 | [-.554, .385] | | |
| Other traumatic SLE [cluster mean centered] | -.107 | .279 | .702 | [-.654, .440] | | |
| Other traumatic SLE [cluster mean centered] * dizygotic | .085^a^ | .240 | .724 | [-.385, .554] | | |
| Age of onset (*t*) | .464 | .087 | < .001 | [.294, .634] | | |
| Age of onset^2^ | -.012 | .004 | .001 | [-.019, -.005] | | |
| Age of onset^3^ | .000 | .000 | .027 | [.000, .000] | | |
| Wave 2 | -.397 | .085 | < .001 | [-.564, -.231] | | |
| Sex (female) | .756 | .127 | < .001 | [.507, 1.004] | | |
| Fixed intercept | -11.878 | .679 | < .001 | [-13.207, -10.548] | | |
| Random part |  |  |  |  |  |  |
| $\sigma_{A}$ | 1.512 | .289 |  | [1.039, 2.200] | | |
| $\sigma_{E}$ | 1.215 | .301 |  | [.748, 1.975] | | |

AIC = 8899.936 (df = 11)

^a^Estimated as single parameter.

**Table A4.**

*AE Model With Economic SLEs and Only Threshold MDE Diagnoses*

| Fixed part | β | *SE* | p | 95% Confidence Interval |  |  |
| --- | --- | --- | --- | --- | --- | --- |
| Economic SLE [cluster mean] | .516 | .238 | .030 | [.049, .983] | | |
| Economic SLE [cluster mean] * dizygotic | .165^a^ | .311 | .595 | [-.445, .775] | | |
| Economic SLE [cluster mean centered] | .861 | .317 | .007 | [.239, 1.484] | | |
| Economic SLE [cluster mean centered] * dizygotic | -.165^a^ | .311 | .595 | [-.775, .445] | | |
| Age of onset (*t*) | .471 | .087 | < .001 | [.301, .640] | | |
| Age of onset^2^ | -.012 | .004 | .001 | [-.019, -.005] | | |
| Age of onset^3^ | .000 | .000 | .023 | [.000, .000] | | |
| Wave 2 | -.401 | .085 | < .001 | [-.568, -.234] | | |
| Sex (female) | .690 | .125 | < .001 | [.445, .935] | | |
| Fixed intercept | -11.854 | .678 | < .001 | [-13.183, -10.524] | | |
| Random part |  |  |  |  |  |  |
| $\sigma_{A}$ | 1.526 | .289 |  | [1.054, 2.211] | | |
| $\sigma_{E}$ | 1.191 | .298 |  | [.729, 1.947] | | |

AIC = 8895.793 (df = 11)

^a^Estimated as single parameter.

**Appendix B**

Appendix B reports the results of descriptive analyses of the temporal relationship between SLE occurrence and MDE onset. A limitation of the current study is that SLE occurrence was only recorded to the nearest year. Previous research has shown that some SLEs may influence depression risk over a shorter time window such as months (Kendler, Karkowski, & Prescott, 1998, 1999).

Figure B1 describes the frequency of time-censored SLEs occurring before MDE onset in years for both W1 and W2. As the plot shows, the frequency of SLE occurrence before MDE generally decreases with increasing years. This implies that fewer SLEs occur when the distance between the year of SLE occurrence and year of MDE onset increases. This is what would be expected in light of previous research, assuming that most SLEs primarily increase risk over a shorter time-period.

This trend of decreasing frequency of SLEs when the temporal distance between SLE occurrence and MDE onset is slightly less pronounced for violent relational SLEs. Although speculative, this could in part be explained by some violent SLEs, such as sexual abuse, which may increase depression risk over a lifetime (Kendler et al., 2000; Kendler & Gardner, 2017).

**Figure B1.**

**Appendix C**

Appendix C reports additional details on the characteristics of the sample. These are based on the sample in the second wave of data collection. We also describe previous studies which have examined the representativeness of the sample below.

***Education***

**Table C1.**

*Education Level in Sample*.

| **Highest achieved education level** | **N (% of total sample size 2288)** |
| --- | --- |
| Primary or middle school, 9 years | 53 (2.32%) |
| High school, 1-2 years | 166 (7.26%) |
| Vocational high school, 3 years | 305 (13.33%) |
| General/economics high school, 3 years | 205 (8.96%) |
| College or university, 4 years or less | 761 (33.26%) |
| College or university, more than 4 years | 753 (32.91%) |
| Doctoral degree | 45 (1.97%) |

***Income***

**Table C2.**

*Income Level in Sample*.

| **Income last year (including all income and benefits)** | **N (% of total sample size 2269)** |
| --- | --- |
| Less than 125.000 | 65 (2.86%) |
| 125.000-200.000 | 135 (5.95%) |
| 200.001-300.000 | 214 (9.43%) |
| 300.001-400.000 | 625 (27.55%) |
| 400.001-550.000 | 730 (32.17%) |
| 550.001-700.000 | 267 (11.77%) |
| 700.001-850.000 | 119 (5.24%) |
| More than 850.000 | 114 (5.02%) |

***Marital status***

**Table C3.**

*Marital Status in Sample*.

| **Marital status** | **N (% of total sample size 2296)** |
| --- | --- |
| Married | 1186 (51.66%) |
| Registered partnership | 28 (1.22%) |
| Cohabitant | 592 (25.78%) |
| Single | 374 (16.29%) |
| Divorced/separated | 114 (4.97%) |
| Widowed | 2 (0.09%) |

Several studies have described the characteristics of the sample used in the current study (Harris, Magnus, & Tambs, 2002, 2006; Nilsen et al., 2013; Tambs et al., 2009). Tambs et al. (2009) examined selection effects and found that participation in the study was associated with being female, having achieved a higher education level and zygosity. Few health-related variables were significant predictors. Furthermore, there were no indications that variables which could influence similarities between twins had significant influence. Tambs et al. (2009) conclude that it is unlikely that recruitment bias, or attrition bias, strongly influenced the sample. The sample characteristics and rationale for the second wave of data collection is also further described by Nilsen et al. (2013).

**Appendix D**

Table D1 reports the total frequency of SLE occurrences in the sample and the number of participants concordant and discordant for time-censored SLE exposure.

**Table D1.**

*Frequencies of Stressful Life Events*

| Stressful life events (SLEs) | Frequency in full sample | Discordant MZ twin pairs | Discordant DZ twin pairs |
| --- | --- | --- | --- |
| Life-threatening illness | 125 | 54 | 32 |
| Life-threatening accident | 151 | 55 | 43 |
| Actively participated in war/combat | 31 | 6 | 12 |
| Witnessed anyone be badly injured or killed | 229 | 63 | 80 |
| Threatened with a weapon, held captive or kidnapped | 153 | 41 | 44 |
| Experienced fire, flooding or natural disaster | 133 | 43 | 42 |
| Rape | 91 | 33 | 29 |
| Sexual abuse | 176 | 51 | 48 |
| Otherwise physically attacked or assaulted | 303 | 93 | 80 |
| Otherwise physically abused as a child | 76 | 20 | 17 |
| Otherwise mistreated as a child | 68 | 13 | 17 |
| Parental mental illness or alcohol problems as a child | 395 | 48 | 50 |
| Parental divorce or separation as a child | 451 | 33 | 14 |
| Having divorced or separated | 709 | 163 | 161 |
| Long-term financial difficulties | 164 | 44 | 40 |
| Unemployment for more than six months | 236 | 60 | 64 |
| Major and lasting conflict with close person | 269 | 66 | 55 |
| Other serious life event | 441 | 134 | 100 |

**References**

Harris, J. R., Magnus, P., & Tambs, K. (2002). The Norwegian Institute of Public Health Twin Panel: A description of the sample and program of research. *Twin Research and Human Genetics*, *5*(5), 415–423. https://doi.org/10.1375/twin.5.5.415

Harris, J. R., Magnus, P., & Tambs, K. (2006). The Norwegian Institute of Public Health Twin Program of Research: An update. *Twin Research and Human Genetics*, *9*(6), 858–864. https://doi.org/10.1375/twin.9.6.858

Kendler, K. S., Bulik, C. M., Silberg, J., Hettema, J. M., Myers, J., & Prescott, C. A. (2000). Childhood sexual abuse and adult psychiatric and substance use disorders in women: An epidemiological and cotwin control analysis. *Archives of General Psychiatry*, *57*(10), 953–959. https://doi.org/10.1001/archpsyc.57.10.953

Kendler, K. S., & Gardner, C. O. (2017). Genetic and environmental influences on last-year major depression in adulthood: A highly heritable stable liability but strong environmental effects on 1-year prevalence. *Psychological Medicine*, *47*(10), 1816–1824. https://doi.org/10.1017/S0033291717000277

Kendler, K. S., Karkowski, L. M., & Prescott, C. A. (1998). Stressful life events and major depression: Risk period, long-term contextual threat, and diagnostic specificity. *The Journal of Nervous and Mental Disease*, *186*(11), 661–669. https://doi.org/10.1097/00005053-199811000-00001

Kendler, K. S., Karkowski, L. M., & Prescott, C. A. (1999). Causal relationship between stressful life events and the onset of major depression. *American Journal of Psychiatry*, *156*(6), 837–841. https://doi.org/10.1176/ajp.156.6.837

Nilsen, T. S., Knudsen, G. P., Gervin, K., Brandt, I., Røysamb, E., Tambs, K., … Harris, J. R. (2013). The Norwegian Twin Registry from a public health perspective: A research update. *Twin Research and Human Genetics*, *16*(1), 285–295. https://doi.org/10.1017/thg.2012.117

Tambs, K., Rønning, T., Prescott, C. A., Kendler, K. S., Reichborn-Kjennerud, T., Torgersen, S., & Harris, J. R. (2009). The Norwegian Institute of Public Health Twin Study of Mental Health: Examining recruitment and attrition bias. *Twin Research and Human Genetics*, *12*(2), 158–168. https://doi.org/10.1375/twin.12.2.158
